# General Relativity, Mental Causation, and Energy Conservation

**DOI:** 10.1007/s10670-020-00284-7

**Published:** 2020-08-04

**Authors:** J. Brian Pitts

**Affiliations:** 1 Faculty of Philosophy and Trinity College, University of Cambridge, Cambridge, UK; 2 School of History and Heritage, University of Lincoln, Lincoln, UK

## Abstract

The conservation of energy and momentum have been viewed as undermining Cartesian mental causation since the 1690s. Modern discussions of the topic tend to use mid-nineteenth century physics, neglecting both locality and Noether’s theorem and its converse. The relevance of General Relativity (GR) has rarely been considered. But a few authors have proposed that the non-localizability of gravitational energy and consequent lack of physically meaningful local conservation laws answers the conservation objection to mental causation: conservation already fails in GR, so there is nothing for minds to violate. This paper is motivated by two ideas. First, one might take seriously the fact that GR formally has an infinity of rigid symmetries of the action and hence, by Noether’s first theorem, an infinity of conserved energies-momenta (thus answering Schrödinger’s 1918 false-negative objection). Second, Sean Carroll has asked (rhetorically) how one should modify the Dirac–Maxwell–Einstein equations to describe mental causation. This paper uses the generalized Bianchi identities to show that General Relativity tends to exclude, not facilitate, such Cartesian mental causation. In the simplest case, Cartesian mental influence must be spatio-temporally constant, and hence 0. The difficulty may diminish for more complicated models. Its persuasiveness is also affected by larger world-view considerations. The new general relativistic objection provides some support for realism about gravitational energy-momentum in GR (taking pseudotensor laws seriously). Such realism also might help to answer an objection to theories of causation involving conserved quantities, because energies-momenta would be conserved even in GR.

## Introduction

The energy conservation objection to nonphysical mental causation has been made starting from Leibniz in the 1600s (Leibniz 1997, 1981, Book I, chapter 1, section 73, 1985, p. 156; Schmaltz 2008, p. 172) until today and has engaged Wolff, Knutzen, Crusius, the young Kant, Maxwell, Helmholtz, Broad (not all on the same side) and many more modern authors. This objection is made with great frequency and often considerable confidence in the contemporary philosophy of mind literature [see citations in Montero (2006), Collins (2008), Gibb (2010), Pitts (2019)] as well as some striking instances (Bunge 1980, p. 17; Dennett 1991, p. 35; Churchland 2011). Even E. J. Lowe thought that conservation laws might be problematic for interactionist dualism, though he proposed several ways out, one of which (though not his favorite) has merit (Lowe 1992, 1996, pp. 56–61, 2003, p. 139; Cucu and Pitts 2019). As various authors have noted, non-epiphenomenalist forms of property dualism suffer from analogous worries about how the mental can affect the physical (Crane 2001, pp. 40, 43, 50; Searle 2004, pp. 44–46; Zimmerman 2007). Thus one needn’t be attracted to substance dualism to encounter this worry: if the deliciousness of pizza is a quale that helps to bring about your eating seconds over and above what your body would have done anyway, then you face the conservation objection. Unfortunately quite a few dualists, not grasping the relevance of the converse of Noether’s first theorem or of the locality of conservation laws in modern physics (Pitts 2019), have tried to argue that mind-to-body causation is compatible with the conservation laws. The converse Noether theorem says that conservation laws imply symmetries, so by contraposition non-symmetries imply non-conservation. Interactionist dualism manifestly violates symmetries, because my soul (if I have one) acts only in my body and not on Mars, and during my lifetime rather than during the Battle of Hastings. Thus non-conservation is a fact about interactionist dualism, at least insofar as physics is described by the principle of least action (which ignores quantum mechanics). But to what extent is such non-conservation an objection rather than a just a consequence that can be accepted?

Recently (Pitts 2019; Cucu and Pitts 2019) this 300+-year-old objection was assessed in light of modern physics, including aspects that rarely come up and some that seem not to have entered the debate at all. It was found that, construed as an argument, the objection begs the question. Such a conclusion is not entirely novel (Watkins 1995; Ducasse 1960; Averill and Keating 1981; Garber 1983; Lowe 1992; Plantinga 2007; Pitts 2020), but evidently it has not been argued in such detail and with an eye to the twentieth century physics of fields. On occasion it has been noted that theoretical physics infers conservation laws from symmetries (Plantinga 2007). This is the topic of Noether’s first theorem and its simpler antecedents. In contrast to Einstein (Gorelik 2002; Pitts 2016b), Max Born, generalizing ideas from Gustav Mie’s electrodynamics and Gustav Herglotz’s relativistic continuum mechanics, had a clear understanding of how rigid translation symmetries imply conservation laws:

The assumption of Mie just emphasized, that the function $\Phi$ [the Lagrangian density] is independent of *x*,  *y*,  *z*,  *t*,  is also the real mathematical reason for the validity of the momentum-energy-law. ...We assert that for these differential equations, a law, analogous to the energy law (3$^{\prime }$) of Lagrangian mechanics, is always valid as soon as one of the 4 coordinates $x_{\alpha }$ does not appear explicitly in $\Phi$ (Born 2007).

This result had nineteenth century antecedents (Lagrange 1997, pp. 233, 234, 1811, p. 318; Hamilton 1834; Jacobi 1996; Kastrup 1987). Clearly interactionist dualist mental causation violates the symmetries of time- and space-translation. My soul (if I have one) acts in my body today, not 500 years ago or on the Moon; your soul (if you have one) is analogous. Thus interactionist mental causation removes the theoretical expectation of conservation by falsifying the symmetry that would otherwise hold. The preceding paper novelly applied the *converse* Noether theorem, which says that conservation laws imply symmetries, to show that one can go further (Pitts 2019). Not only is there no reason to expect conservation to hold, but there *is reason to expect it not to hold*, given interactionism. Hence given the dialectic of the attempted objection to interactionism, the critic needs to give some reason for expecting conservation to hold *in the brain* that *has some purchase on the interactionist*. But there is apparently none to be had, at least not from physics (as opposed to empirical neuroscience), or at any rate not from classical field theories excluding General Relativity. Hence the conservation argument against interactionism degenerates into a mere denial or an incredulous stare. If philosophers of mind aim to respect real physics rather than A-level chemistry (a concern previously directed toward metaphysicians (Ladyman et al. 2007, p. 24), then it is necessary to notice that in view of Noether’s first theorem and its converse, physics implies a biconditional rather than categorical status of conservation laws. Hence the traditional Leibniz-to-Dennett energy conservation objection fails. One of course can and should employ neuroscience *a posteriori* for more serious objections to interactionist dualism, but that is a completely different argument, one having only accidental connections to conservation laws.

The failure of the Leibniz-to-Churchland energy conservation objection is not really news to philosophers of physics. As Jeremy Butterfield wrote over two decades ago:

...[A] traditional argument against interactionism is flawed....The idea is that any causal interaction between mind and matter would violate the principle of the conservation of energy....But, says the argument, physics tells us that energy is conserved in the sense that the energy of an isolated system is constant, neither increasing nor decreasing....And there is no evidence of such energy gains or losses in brains. So much the worse, it seems, for interactionism. [Though traditional, the argument is still current; for example, Dennett endorses it (1991, pp. 34–35).]

This argument is flawed, for two reasons. The first reason is obvious: who knows how small, or in some other way hard to measure, these energy gains or losses in brains might be? Agreed, this reason is weak: clearly, the onus is on the interactionist to argue that they could be small, and indeed are likely to be small. But the second reason is more interesting, and returns us to the danger of assuming that physics is cumulative. Namely: the principle of the conservation of energy is not sacrosanct. ...[A]lthough no violations have been established hitherto, it has been seriously questioned on several occasions. It was questioned twice at the inception of quantum theory.... And furthermore, it is not obeyed by a current proposal ...for solving quantum theory’s measurement problem.

In short: physicalists need to be wary of bad reasons to think physicalism is true, arising from naivety about physics (Butterfield 1997).

Note that Butterfield, who is not himself sympathetic to dualism, doesn’t seriously entertain the idea, common in the philosophy of mind, that the failure of exact conservation constitutes an interesting objection. Of course conservation fails, given that it follows from symmetry and the symmetry is violated by interactionism. (The symmetry-to-conservation law link in Noether’s theorem is common knowledge among philosophers of physics, though the converse was perhaps less well known until recently.) A more interesting question for interactionists is whether their view violates conservation laws badly enough to run afoul of experiment. Unfortunately Butterfield’s insights have not had the deserved effect on the philosophy of mind literature.

The failure of the traditional Leibnizian objection occurs in what would seem to be the most conservation-friendly territory available, classical local field theory with the principle of least action (Pitts 2019).^1^ Aspects of conservation laws in modern physics that have been largely overlooked in the philosophy of mind literature include some mentioned above and others as well: locality (energy conservation holds not primarily for the whole universe, but in every place separately) (Lange 2002, ch. 5), conditionality upon the absence of external influences, indeed biconditionality (symmetries and conservation laws being mutually entailing by Noether’s first theorem and its converse (Noether 1918; Brading 2001; Brown and Holland 2004; Kosmann-Schwarzbach 2011; Romero-Maltrana 2015; Goldstein 1980, chapter 12), and gentle failure (robustness implying that a small violation of conservation would not be catastrophic, in line with Butterfield (1997) and contrary to Bunge (1980)). Basically the same holds for the conservation of momentum. Thus Noether’s theorem leaves the door open to mental causation; the traditional Leibnizian energy conservation objection simply assumes what needed to be proven. Hence any quantum-based replies to the objection that one might give (as many do) push at an already open door (unless General Relativity closes the door), though possibly it doesn’t hurt to have additional replies anyway, especially for a widely dismissed view. Of course anyone aiming to give a *positive account or a defense* of traditional mind-body interaction (which I do not aim to do) might need to take into account quantum physics somehow. Given that this paper explains how General Relativity implies a less trivial objection than the traditional Leibnizian one, quantum replies could become relevant for that reason also.

The mind-body problem was of considerable interest to Herbert Feigl (1958, 2003, 2004). Feigl took the conservation of energy to be only empirically valid and potentially subject to refutation (Feigl 1958, p. 472), so he (wisely) would be unlikely to make the Leibnizian energy conservation objection to interactionism. Presumably he and other logical empiricists would have been interested in the bearing of General Relativity on the philosophy of mind (if there is one) and likely would have been pleased to find therein some evidence against interactionism.

### Enter General Relativity

What difference does General Relativity make? While General Relativity is by now over a century old, there seems to have been no correct exploration of its bearing on the conservation law issue in the philosophy of mind until now. Typically the energy conservation objection is based on a rather elementary grasp of physics, roughly high-school chemistry level, corresponding perhaps to the physics of the 1860s, so any attempt to address the difference made by General Relativity is welcome.

Two better-informed authors have claimed that conservation laws already fail in General Relativity even apart from dualism (Mohrhoff 1997, 1999; Collins 2008, 2011); thus there is no conservation law left for dualism to violate, so the usual objection is eliminated. Thus they invoke not a Noether biconditional relation between symmetries and conservation, but General Relativity’s supposed lack of conservation laws to respond to the Leibnizian objection. The Mohrhoff–Collins invocation of General Relativity is, doubtless, a rhetorically impressive move: by knowing much more physics than usual, one supposedly shows that the usual, supposedly scientific objection, fails. Surprisingly to those outside the field of General Relativity, it is unusual to take the formal conservation laws and gravitational energy seriously in General Relativity. The Mohrhoff–Collins proposal takes that widely shared idea in the General Relativity literature and exploits it for the philosophy of mind.^2^ Relatedly, Roger Penrose has proposed that the energy non-conservation difficulty of GRW spontaneous collapse quantum theory might find resolution in some future framework through linkage to the gravitational energy nonlocalization (Penrose 1994, pp. 334, 344–346); this proposal also has links to the philosophy of mind.

Generally there is something of an anti-realist tendency regarding energy conservation in General Relativity, at least regarding the local conservation laws, the kind that elsewhere modern physics usually employs. An old standard textbook decreed that “[a]nybody who looks for a magic formula for ‘local gravitational energy-momentum’ is looking for the right answer to the wrong question.” (Misner et al. 1973, p. 467) If gravitational energy isn’t really anywhere in particular, then non-gravitational energy doesn’t satisfy an honest conservation law, either, because of interconversion between material energy and gravitational (pseudo-?)energy. Instead one has only a balance law $\nabla _{\mu } (\sqrt{-g} T^{\mu \nu }) = \partial _{\mu }(\sqrt{-g} T^{\mu \nu }) + \sqrt{-g} T^{\mu \alpha }\Gamma _{\mu \alpha }^{\nu } =0$ describing how non-gravitational energy fails to be conserved due to gravitational influence. This balance equation, because of the second term, typically does not imply any global conservation law (Weyl 1922, pp. 236, 269–271; Landau and Lifshitz 1975, p. 280; Misner et al. 1973, p. 465; Lord 1976, p. 139; Stephani 1990, p. 141). While most physicists now accept that gravitational energy exists [which has not always been the case (Kennefick 2007)], it is difficult to make sense of this nonlocalizability, an apparent lack of any objective location. In recent decades some philosophers have sought conceptual clarity by denying the existence of gravitational energy (Hoefer 2000; Duerr 2019a), which seems to have incompatible properties such as being present and absent at the same space-time point.

But if one is a realist about gravitational energy localization and conservation, one will be quite disinclined to accept the Mohrhoff–Collins move. General Relativity has uncountably infinitely many symmetries of the action^3^ and thus, at least formally, infinitely many conserved currents (Bergmann 1958, 1992; Kijowski 1978; Szabados 2009). The gravitational energy realist takes this mathematics seriously and infers that General Relativity is *more* conserving of energy than other theories, not less so, so broadly Cartesian mental causation should be harder, not easier, given General Relativity (Pitts 2010). The fact that Einstein’s equations *alone*, without help from the matter field equations, entail conservation laws (Noether 1918; Bergmann 1958; Pitts 2010, 2016a; Anderson 1967, p. 428) is further evidence that General Relativity is unusually supportive of conservation laws rather than unusually lax as is often claimed. But is one left with a choice of a common non-realist interpretation and a less popular realist interpretation, with no means of definitive adjudication for the consequences for mental causation?

Fortunately there *is* a way to resolve the question of the bearing on mental causation objectively, one of the sort that Leibniz himself would have approved: “Calculemus!” One can do new calculations involving the generalized Bianchi identities, without taking a prior interpretive stand on the controversies involving gravitational energy. These new calculations, being tensorial, are entirely free of the confusion and controversy that have swirled about gravitational energy for a century due to the perplexing features of pseudotensors (which have at least the virtue of being motivated by symmetries of the action in accord with Noether’s theorem) and the difficulty of finding an adequate alternative. When one does the generalized Bianchi identity mathematics (below), one finds that General Relativity makes Cartesian mental causation *harder*, not easier. Thus one can go beyond interpretive stances and achieve objective results.

In the simplest case, when the Cartesian mental influence is a scalar field (a single number at each point, the same in all coordinate systems), that influence must be spatio-temporally constant, as will appear below. Spatio-temporal constancy is obviously absurd for the influence of a human mind unless that influence is zero, so that there is no Cartesian mental causation after all. Unlike the traditional Leibnizian objection, this new objection from General Relativity does not assume something equivalent (given Noether’s theorem and its converse) to non-interaction in order to infer non-interaction. Thus General Relativity resists external influences by trying to force them to vanish. While the simplest toy model of mind-body influence yields a negative result for the ability of the non-physical mind to influence the body, this result weakens as the complexity of the model rises. The complexity of the model for mental causation is also bound up with larger world-view issues, as will appear below. Because the majority (non-localization) interpretation of gravitational energy has the wrong heuristic force, leading one to think that General Relativity is more lax when in fact it is more rigid, the non-localization interpretation is somewhat undermined by the objective Bianchi identity results. Correspondingly, the minority realist interpretation has the correct heuristic force and so is somewhat confirmed.

Realism about gravitational energy is philosophically interesting for another reason, namely, the relevance to conserved quantity theories of causation (Fair 1979; Dowe 2000b). A number of authors have recognized that the conventional doubts about the reality of localized gravitational energy and the consequent lack of true conservation laws pose problems for regarding energy and momentum as conserved quantities, thus depriving conserved quantity theories of causation of their star examples (Rueger 1998; Dowe 2000a; Curiel 2000; Lam 2010; Vassallo 2020). If energy and momentum are not conserved quantities, then conserved quantity theories of causation seem quite hopeless. Realism about gravitational energy localization and hence the pseudotensorial local conservation laws would imply that General Relativity potentially no longer poses an objection to theories of causation that take energy to be a conserved quantity. One should, however, get accustomed to making energy plural (conserved quantit*ies* theory of causation), because the mathematics speaks of infinitely many conserved energies and momenta if it speaks of any. One might see the help given to conserved quantity theories of causation as another theoretical virtue that counts somewhat in favor of realism about gravitational energy(s). But there are lingering difficulties not unrelated to those that motivated doubts about the reality of gravitational energy in the first place.

In arguing for “realism” about gravitational energy, I do not intend to be making novel moves in metaphysics, but rather to be taking for granted the presumably unproblematic (at any rate rarely criticized) status of non-gravitational energy in other theories and arguing that gravitational energy has the *same* status as non-gravitational energy. If one already doubts the existence of non-gravitational energy, or if one comes to doubt its existence by being persuaded that non-gravitational energy is no better than gravitational energy, such views would be perfectly compatible with the views defended here. Given that most people seem content with non-gravitational energy and that I do not object to this view, I will continue to describe my view of gravitational energy as realist despite only arguing for parity between non-gravitational energy and gravitational energy.

## Mental Causation: Carroll’s Foundling Program

The question whether the causal influence of immaterial minds (or for that matter, nonredundantly causal mental properties) can be modelled in physics, though not novel, has become the more timely in light of the recent interventions of Sean Carroll, Caltech cosmologist, amateur philosopher, and proponent of “poetic naturalism,” which one can choose as one’s religion at Facebook (Carroll 2018). Judging by Carroll’s rhetoric, the answer is clearly negative, apparently apart from calculations, literature searches, or experiments. Nonphysical mental causation would “overthrow everything we think we have learned about modern physics” (Carroll 2011). Readers versed in physics might be startled to hear that, say, the spin-statistics theorem (that one should quantize bosonic fields using commutators and fermionic fields using anticommutators, a standard result of quantum field theory (e.g., Kaku 1993, p. 88; Peskin and Schroeder 1995, pp. 52–56), or the apparent empirical adequacy of representing all spatio-temporal-gravitational physics in terms of a single space-time metric tensor, would be overthrown by immaterial souls with causal influence. Lay readers, on the other hand, might be inclined to accept Carroll’s claim.

Carroll’s recent semi-popular philosophy book (Carroll 2016, pp. 212, 435–441), which purports to derive science-based conclusions for great questions including the philosophy of mind and the question of life after death, has been reviewed in *Science*. Where the book discusses the bearing of physics on the philosophy of mind, it continues the earlier program. Carroll’s rhetorical questions can serve to inspire a genuine inquiry into his questions. It turns that whereas the traditional Leibnizian energy conservation objection is question-begging (Pitts 2019), Carroll raises a good question in asking how, if at all, physics could be modified so as to take account of the influence of immaterial minds on the electrons in brains. In teaching a wide audience about “Physics and the Immortality of the Soul,” Carroll displays the Dirac equation, though he later claims that the mere existence of the equation, not its detailed form, matters. His informal argument against dualism and immortality follows.

As far as every experiment ever done is concerned, this equation is the *correct* description of how electrons behave at everyday energies. It’s not a complete description; we haven’t included the weak nuclear force, or couplings to hypothetical particles like the Higgs boson. But that’s okay, since those are only important at high energies and/or short distances, very far from the regime of relevance to the human brain.

If you believe in an immaterial soul that interacts with our bodies, you need to believe that this equation *is not right*, even at everyday energies. There needs to be a new term (at minimum) on the right, representing how the soul interacts with electrons. (If that term doesn’t exist, electrons will just go on their way as if there weren’t any soul at all, and then what’s the point?) So any respectable scientist who took this idea seriously would be asking—what form does that interaction take? Is it local in spacetime? Does the soul respect gauge invariance and Lorentz invariance? Does the soul have a Hamiltonian? Do the interactions preserve unitarity and conservation of information?

Nobody ever asks these questions out loud, possibly because of how silly they sound. Once you start asking them, the choice you are faced with becomes clear: either overthrow everything we think we have learned about modern physics, or distrust the stew of religious accounts/unreliable testimony/wishful thinking that makes people believe in the possibility of life after death. It’s not a difficult decision, as scientific theory-choice goes (Carroll 2011, emphasis in the original).

The careful reader will notice that Carroll looks for evidence only to experiments aimed at testing how electrons, electromagnetism and gravity interact, ignoring the possibility that a new term on the right could vanish in the apparatus at SLAC, Fermilab, CERN and the JINR, while being nonzero elsewhere, such as in brains (Thompson 2008), or perhaps on occasion in monasteries, churches, ashrams, séances, or the like—places that spirits, if they exist and act on matter, might find especially relevant. Brian Cox’s claim that the Large Hadron Collider has disproven ghosts (Pomeroy 2017) is flawed for the same reason: ghosts, if they exist, presumably are more likely to appear at haunted houses, séances, and the like than in particular accelerators. (The contrary view, perhaps animistic, that ghosts are just as likely in one place as another, is indeed disconfirmed by the success of particle physics. But if ghosts are persons, they might have reasons for favoring some places over others. They haven’t visited me, so I cannot be sure.)

The crucial undefended randomizing assumption that mind- or parapsychology-relevant violations of energy conservation are just as likely to be in one place as another was at least explicitly *stated* by W. K. Clifford:

It has been supposed, I say, by some people, as it seems to me merely by a confusion of ideas, that there is, at some part or other of this process, a creation of energy ; but there is no reason whatever why we should suppose this. The difficulty in proving a negative in these cases is similar to that in proving a negative about anything which exists on the other side of the moon. It is quite true that I am not absolutely certain that the law of the conservation of energy is exactly true ; but *there is no more reason why I should suppose a particular exception to occur in the brain than anywhere else*. I might just as well assert that whenever anything passes over the Line, when it goes from the north side of the Equator to the south, there is a certain creation of energy, as that there is a creation of energy in the brain. If I chose to say that the amount was so small that none of our present measurements could appreciate it, it would be difficult or indeed impossible for anybody to disprove that assertion ; but I should have no reason whatever for making it. There being, then, an absence of positive evidence that the conditions are exceptional, the reasons which lead us to assert that there is no loss of energy in organic any more than in inorganic bodies are absolutely overwhelming (Clifford 1874, emphasis added).

Whether Clifford’s assumption (tacit also in Carroll and Cox) is plausible depends entirely on which of two closely related but distinct targets one has in mind: spirits or parapsychology-as-subtle-physics. It is especially easy to conflate these two targets due to the gradual shift (Cerullo 1982; Asprem 2010) from spiritualism to psychical research (with tensions between spiritualist and more scientific wings) to clearly *methodologically* scientific experimental parapsychology. To my knowledge most interactionist dualists think that souls act on brains, or perhaps on bodies as a whole, but not on the rest of the physical world. Proponents of psychokinesis, rare but not nonexistent among parapsychologists, might demur from this limitation (Owen 1964, 1975; Owen et al. 1975; Braude 1986; Josephson 2004; Grosso 2016); but such claims are purportedly scientific and not paradigmatically religious or traditional dualist claims. Carroll and Cox evidently envisage that psychic powers, souls, *etc.* must be envisaged as a sort of subtle physics, implemented *via* new physical particles/fields or the like. There are, of course, people who believe precisely this. One can find views in this neighborhood in some of the most visible work in parapsychology (Cardeña et al. 2015; Grosso 2016).^4^ I largely agree with Carroll’s critique: if there were physical fields/particles that mediated psychic powers, experimental physics ought to have discovered them, but it hasn’t, so there are almost certainly not any such things.

The problem is that Carroll claims to have shown something else, not merely that there isn’t some subtle physics explaining paranormal powers, but that there are no souls acting on bodies and (therefore?) no life after death (Carroll 2016, p. 4, chs. 19–24). Yet his critique entirely fails if such events, powers, *etc.* are implemented *via* non-physical personal entities: immaterial souls, God, angels, demons, genies, ghosts, or the like. Being non-physical and personal, such influences are not equally available and testable everywhere and always, *pace* Clifford; rather, they are available and testable (if they exist) only in particular space-time regions—in living brains, around saints, in prayer meetings, at séances, near witchdoctors (Young and Goulet 1994), or the like. Brains, at least, are rather reliably available, but still sufficiently localized that experiments on thigh muscles or in nuclear physics laboratories are irrelevant (a point overlooked surprisingly often). By extrapolating from experimental physics and ignoring the potential disanalogies introduced by the spiritual context (at least as judged by some of the people whose views he claims to refute), Carroll has generated a conflict between such testimonies and experimental physics. Given that he is on the offensive dialectically, his argument begs the question.

The point that evidence for anomalous events needs to be sought *where and when such events allegedly happen(ed)*, not where one finds it convenient to look, has been made before, such as in the eighteenth century by defenders of miracles Gottfried Less, George Campbell and William Paley (Craig 1986). Otherwise one is like the proverbial intoxicated man who looks for his keys under the street light rather than where he probably dropped them (Anonymous 2019); thus Clifford in effect endorsed the drunkard’s search principle, whereas Less, Campbell and Paley rejected it. Miracles and soul-to-body causation, if they happen at all, are not uniformly distributed events. Evidence that such events have occurred in certain times and places does not contradict evidence that they haven’t occurred elsewhere. If there is evidence that such events have happened, then there is evidence that the allegedly universal law isn’t a universal law, and there is no conflict between the evidence for the particular exception and the supposedly stronger evidence for the universal law. Perhaps Hume’s critique of miracles underlies Carroll’s reasoning, but that is hardly an uncontested argument (Earman 2000) and is clearly philosophical rather than scientific.

Readers versed in the philosophy of mind might think that an argument from the (supposed) failure of interactionist dualism to the denial of an afterlife is a bit quick; one might consider such presently even less fashionable substance dualist options as occasionalism, pre-established harmony, and epiphenomenalism before discarding an afterlife on the basis of troubles with interactionism; perhaps idealism is also worth another look. Given that my purpose here is not to defend ideas of an afterlife but to develop Carroll’s abandoned program of evaluating interactionism physically, I will not pursue such questions further, however.

Carroll’s claims about the bearing of contemporary physics on interactionism are controversial.

How an immaterial soul might interact with the physical body remains a challenging question for dualists even today, and indeed it has grown enormously more difficult to see how it might be addressed [compared to the days when Elisabeth queried Descartes]....(Carroll 2016, pp. 212),

But in fact a fair number of serious people have suggested that such interaction is in fact made *easier* rather than harder, due to quantum mechanics (Eccles 1994; Atmanspacher 2015). Wigner took quantum mechanics to provide reason to infer mental influence on the physical world (Wigner 1962). Such claims are not merely from decades ago; there are recent proposals from Halvorson (2011) and Kent (2018). I set such claims aside, however, because I am interested in ways that modern physics might make such mental conservation harder rather than easier.

One might be tempted to take Carroll’s claim to be akin to those of (mostly) physicalist philosophers of mind who raise the energy conservation issue, but in fact there are key differences. For one, Carroll doesn’t bring up energy conservation [at least not here, though eventually he does elsewhere (Carroll 2016, p. 356)]. Perhaps for a theoretical physicist, energy conservation would be too obviously question-begging an objection to mention, given the connection between symmetries and conservation laws? Or perhaps Carroll’s interpreting General Relativity as not having energy and momentum conservation laws (Carroll 2010, 2004) explains his failure to list it as a casualty of soulish causal influence. Elsewhere he claims that the total energy of the universe is 0 in light of General Relativity (Carroll 2016, p. 201), a claim that seems to backpeddle from that strong anti-realism.

Carroll performs dualists a service by clearly formulating the issue and sketching a potentially illuminating research program. Indeed much of the *philosophical* worry about interactionism, such as Princess Elisabeth’s understandable inability to conceive how such disparate entities as mind *with no spatial location* and matter could interact, already starts to dissipate upon being framed in contemporary physics. While it has sometimes been unclear what this objection is at least in modern philosophical reiterations (Kim 2003; Burge 2007; Wong 2007), formulation in modern physics makes the objection even less intelligible. Interactions happen by terms in equations of motion; two fields interact, such as the electromagnetic field and the electron field, if their Lagrangian density a term involving products of both fields (and/or their derivatives), so that each field appears in the other’s equation of motion (Kaku 1993; Peskin and Schroeder 1995). This fact, as it happens, makes something that naively seemed highly implausible—that something could be real and yet invisible (due to not coupling to the electromagnetic field)—now quite pedestrian, as the search for dark matter highlights. Likewise there is no obvious difficulty in having two objects occupy the same space, if one is made of one collection of fields, the other is made of another collection of fields, and the two collections of fields have no mutual interaction terms in the Lagrangian. (So it is at least in *classical* field theory; quantum field theory has more the flavor that what is possible soon becomes necessary.) What, then, is to stop an immaterial substance from having effects that appear in a Lagrangian? It seems that there is just nothing much else to say in response to “how?” unless one rejects the usual standards of modern physics. The de-materialization of everything including matter into fields, suggested around 1930 by Pascual Jordan and by now generally accepted in physics, has left the contemporary understanding of the physical world far more ethereal (so to speak), ghostly, tenuous, and mathematical than either the seventeenth century mechanical philosophy or tables-and-chairs daily experience would have suggested. Carroll is not worried about spatially non-located souls, because he wants to refute the following view:

Very roughly speaking, when most people think about an immaterial soul that persists after death, they have in mind some sort of blob of spirit energy that takes up residence near our brain, and drives around our body like a soccer mom driving an SUV (Carroll 2011).

Hence the greatest source of difficulty faced by Princess Elisabeth, nonspatiality of souls, does not concern Carroll. After conceiving an interesting research program, the implementation of which might in fact tend to undermine interactionist dualism, Carroll abandoned it. I, however, will take it up, at least in part, thereby finding a new objection to interactionist dualism from General Relativity. Carroll’s foundling research program suggests exploring what could possibly be the “new term (at minimum) on the right, representing how the soul interacts with electrons” to assess the prospects for modeling soul-brain interaction in a fashion congenial to theoretical physicists. Such a term would, if all goes well for the dualist, preserve core physical principles insofar as one could reasonably expect (such as locality, gauge invariance, *formal* Lorentz invariance (though obviously violating all the symmetries *de facto* in those spatio-temporal regions where the soul acts on the brain), and unitarity (conserved positive normalized probabilities). I aim for something less ambitious, namely to see what bearing General Relativity has on the energy conservation question. Happily for Carroll and unhappily for interactionist dualists, it turns out that there *is* a difficulty in the vicinity.

To try to write down a local field interaction broadly consonant with modern physics, it is natural to treat souls as spatially located and extended, denying some aspects of the Cartesian view asserted by Foster (2001) and criticized by Fodor (1998). Spatially located souls are both mildly fashionable nowadays [at least as souls go (Eccles 1987; Hasker 2001; Zimmerman 2007)] and closer to the historical mainstream than one might have thought (Vailati 1993; Zimmerman 2007; Reid 2008; Brown 2012). “For even if philosophers today very often take for granted that immaterial entities have no location, this is in fact quite an extraordinary view, historically speaking” (Pasnau 2011, p. 328). Thus one naturally doubts one of Fodor’s assumptions about dualism: “If the mind is nonphysical, it has no position in physical space. How, then, can a mental cause give rise to a behavioral effect that has a position in space?” (Fodor 1998) Indeed Lycan has advised dualists to give up nonspatiality (Lycan 2009):

The big problem for interaction is and remains the utter nonspatiality of Cartesian egos. (By now we can all tolerate action at a distance. But action at a distance is at least at a distance.) My suggestion is that the dualist give up nonspatiality. Descartes had his own seventeenth-century metaphysical reasons for insisting that minds are entirely nonspatial, but we need not accept those. Why not suppose that minds are located where it feels as if they are located, in the head behind the eyes? [footnote suppressed] (Lycan 2009)

Spatial extension also leaves more room for efforts toward detailed dualist neuroscientific proposals, such as Eccles made (Popper and Eccles 1983; Eccles 1994; Smith 2001), some of which have been criticized (Clarke 2014). Jaegwon Kim briefly entertained the idea that spatially locating souls might help to address his pairing problem (Kim 2003, section V).

## Quantum Field Theory in Curved Space-Time as a Precedent

The subject of quantum field theory in curved space-time provides a relevant precedent for assessing Carroll’s claim that soul-to-body influence would “overthrow everything we think we have learned about modern physics....” He elsewhere says:

The Core Theory of contemporary physics describes the atoms and forces that constitute our brains and bodies in exquisite detail, in terms of a rigid and unforgiving set of formal equations that leaves no wiggle room for intervention by nonmaterial influences” (Carroll 2016, p. 212).

It is difficult to know what such wiggle room would be such that modern physics lacks it. Is the problem that modern physics is deterministic? Of course it isn’t clear that modern physics is deterministic—that depends on one’s interpretation of quantum mechanics and even on one’s interpretation of gauge freedom [though everyone manages to arrive at basically the same place on the latter issue, even if by different routes (Butterfield 1989)]—but it is clearly no more deterministic than Newton physics. But even deterministic physics as such presents no obstacle. If there are causally active souls, presumably they introduce novel forces into the laws and make the equations different from what they would have been without causally active souls. That the equations of motion in the absence of soulish influence are deterministic is irrelevant, because those equations would not apply to the physical world in case souls act.

What about rigid and unforgiving equations? Physicists write books on how to do quantum field theory in the presence of spatio-temporally varying influence: quantum field theory in curved space-time (Birrell and Davies 1982; Fulling 1989; Wald 1994; DeWitt 2003; Bastianelli and van Nieuwenhuizen 2006; Parker and Toms 2009; Hack 2016), and the answer is not that everything goes splat and all physical knowledge is lost.^5^ The soul’s influence, in varying spatio-temporally and lacking symmetries, is not altogether unlike the influence of curved space-time, a sort of external potential, on quantum fields. Indeed one might think that souls’ influence would be far less disruptive in some respects, because souls’ influence would only violate time- and space-translation symmetries in the few modest spatio-temporal regions where souls allegedly act, while leaving intact enough idea of the action of the Poincaré group to define particles, for example. One might hazard some conjectural answers to Carroll’s rhetorical questions: “what form does that interaction take? Is it local in spacetime?” Presumably so to the latter question. “Does the soul respect gauge invariance and Lorentz invariance?” Presumably, yes and (excepting the obvious sense of violating the translation symmetries in some localized spatio-temporal regions) yes. “Does the soul have a Hamiltonian?” No, but its *effects* should figure in a modified action principle for physical fields. “Do the interactions preserve unitarity and conservation of information?” Presumably they do as much as physics needs. [He provides an additional list of useful questions in the book (Carroll 2016, p. 216).] Carroll’s claim that souls acting on bodies would “overthrow everything we think we have learned about modern physics” (Carroll 2011) is epistemically possible. However, such a conclusion should be not a bare assertion for mass consumption, but the result of detailed consideration of similarities and dissimilarities between soulish influence and gravitational influence in the context of quantum field theory in curved space-time, an extant theory of quantum fields interacting with an asymmetrical external potential. The next section shows how one *can* pursue analogously detailed questions in the context of classical gravity and how the results are somewhat congenial to Carroll’s poetic naturalist religious project, at least if one supplements or replaces his treatment of conservation laws in General Relativity. In General Relativity one does find a somewhat rigid and unforgiving set of formal equations at last—although this rigidity is finite. If one were attempting to grope towards biological realism, one would, as Carroll notes, need to have the soul interact especially with electromagnetism and electrons, but that is not my aim.

## Does General Relativity Help Mental Causation?

According to Eddington, the Archbishop of Canterbury once asked Einstein about the relevance of relativistic physics for religion; Einstein replied that relativity was a purely scientific theory and had no relevance to religion (Eddington 1939, p. 7). Those who have suggested that General Relativity provides evidence for creation *ex nihilo* might disagree with Einstein; doubts about such claims have been expressed, however [e.g., (Pitts 2008)]. But gravitational energy is another area where General Relativity might have some bearing on religion, whether for good or for ill. As appeared above, a couple of authors have claimed that General Relativity makes it easier for souls to act on bodies (Mohrhoff 1997, 1999; Collins 2008, 2011). Such a claim, if true, would make General Relativity positively relevant to religion.

However, an early result of attempting to carry out Carroll’s foundling program to model the causal influence of immaterial minds on the material world turns out to yield a novel *objection* to such causal influence from General Relativity. This objection naturally involves delving into the topic of gravitational energy and general relativistic conservation laws. General relativistic conservation laws have been a thicket of controversy for a century, though philosophers have rarely discussed the subject until recently [it now being a hot topic (Russell 1927, p. 86; Graves 1971; Rueger 1998; Curiel 2000; Hoefer 2000; Collins 2008, 2011; Pitts 2010; Lam 2010, 2011; Dewar and Weatherall 2018; Read 2018; Duerr 2019a)].

Mohrhoff and Collins (independently) follow a very common interpretation in the physics and philosophy of physics communities (Misner et al. 1973, p. 467) that denies the physical meaning of the formal mathematics of “pseudotensor” conservation laws in General Relativity. The orthodox view in General Relativity for some time has been, to recall from above, that “[a]nybody who looks for a magic formula for ‘local gravitational energy-momentum’ is looking for the right answer to the wrong question” (Misner et al. 1973, p. 467). Mohrhoff and Collins, unlike others writing in the philosophy of mind, were aware of this standard view about General Relativity. One can compare their comparatively well informed view to what was intended as an uncontroversial remark deferential to standard physics of conservation laws:

...about as well established as anything could be in physics, the conservation of mass and energy tells us that in a “closed” system changing over time, the net total of mass or energy in the system, stays the same (Westphal 2016, p. 41).

By contrast Mohrhoff and Collins knew the situation in the General Relativity literature, and then proceeded to take it seriously in the philosophy of mind, thus finding a loophole useful for dualists (Mohrhoff 1997, 1999; Collins 2008, 2011). The issue with gravitational energy is that gravitational energy-momentum depends in a peculiar and *essential* way on the choice of coordinate description, which presumably physically real quantities would not do. That is, gravitational energy-momentum is not described by a “geometric object” (Bergmann 1958; Anderson 1967, pp. 427–430), a set of components relative to each local coordinate system and a transformation rule relating coordinate systems where both apply (Schouten 1954; Nijenhuis 1952). One can have energy absent when intuitively it ought to be present (as Schrödinger noted) or energy present when intuitively it ought to be absent (as Bauer noted) (Schrödinger 1918; Bauer 1918; Cattani and De Maria 1993), at least using Einstein’s canonical pseudotensor, and presumably using the other pseudotensors as well. Thus Schrödinger’s objection is that there is a false negative (no energy outside a heavy body), whereas Bauer’s objection is that there is a false positive (energy, indeed infinitely much energy, in empty flat space-time).

The prolonged absence of any sensible physical interpretation of the general relativistic conservation laws has encouraged a widespread view that any local notions of conservation or spatial presence of gravitational energies lack objective physical significance. There was even a time in the 1930s and 1950s when some leading general relativists (at times including Einstein) doubted the existence of gravitational waves and/or gravitational energy altogether, although this view largely disappeared due to the late 1950s sticky bead argument that gravitational wave energy could be converted to heat (Bondi 1957; Feynman 1971; Kennefick 2007). (Recently the denial of gravitational energy has been revived (Hoefer 2000; Duerr 2019a), a principled view perhaps more plausible than the orthodox one. Relatedly, Cooperstock has long argued that there is no gravitational energy outside matter and hence no energy in gravitational waves (Cooperstock 2000).] Hence there are no local conservation laws, notwithstanding (it is said) the impression given by local pseudotensor conservation laws. There is some resistance to such claims, however, including new ideas for dealing with the two main problems, the non-uniqueness (Chang et al. 1999) and coordinate dependence (Pitts 2010) of pseudotensors; in both cases there is suggestion that multiplicity is a meaningful virtue rather than a defect. Peter Bergmann once indicated that, roughly speaking, these two infinities are in fact one and the same infinity (Bergmann 1958). Authors interested in applications tend to find gravitational energy useful (Petrov and Katz 2002).

## Local Conservation in General Relativity?

One reason that General Relativity makes the mind-body problem harder builds on the mathematical fact that General Relativity not only entails local conservation laws (as do other theories, though they require help from matter equations of motion, unlike General Relativity), but also (unusually) is entailed by conservation laws (Noether 1918; Bergmann 1958; Pitts 2010, 2016a; Anderson 1967, p. 428). Descartes’s effort to found physics on conservation laws (Belot 2006) is finally realized. The mathematics is old and uncontroversial, even if somewhat forgotten.

My view is distinctive in proposing that this mathematics can be taken seriously, that is, realistically. [Nester and collaborators make somewhat similar claims (Chang et al. 1999), though they are mostly interested in interpreting the nonuniqueness of pseudotensors and in pointing out that pseudotensors, considered bad and passé, are quasi-local, a property considered good and modern. Nester elsewhere admits to not following the advice of his teacher, one of the authors of Misner et al. (1973), p. 467, Nester (2004).] General Relativity has uncountably infinitely many time translation symmetries of its Lagrangian (Bergmann 1958): one definition of being at rest (part of a vector field with components (1, 0, 0, 0) in some coordinates, at least in a neighborhood) is equivalent to another definition of moving in an irregular way (that same vector field with wiggly components in some other coordinate system).^6^ By Noether’s first theorem, such a symmetry as time translation (which the first definition exemplifies) implies a conservation law (of energy). But this vector field is itself arbitrary, apart from being non-zero and time-like (at least in a neighborhood). Thus formally one has infinitely many conserved energies. This claim is rarely considered [but see (Bergmann 1958; Kijowski 1978; Szabados 1991)] and is usually considered paradoxical if it is considered at all. I propose that the paradox is due to a tacit and unargued Highlander-type assumption that “there can be only one” energy.^7^ Why can’t all the energies be real (Pitts 2009b, 2010)? It is a bit odd to suggest that a theory logically equivalent to infinitely many (formal) conservation laws, which exist thanks to infinitely many translation symmetries of the Lagrangian (in light of Noether’s theorem), in fact lacks any conservation laws at all, in effect setting $\infty =0$. Just because it’s infinite doesn’t mean it’s 0 (to recall a phrase used around quantum field theory in the late 1940s). If one embraces infinitely many energies, then the Noether symmetry-conservation link resurfaces and the counting works: $\infty =\infty$.

A linguistic analogy will illustrate why pseudotensorial behavior could be a virtue. Suppose that one is learning about Mary/María and encounters the English sentence “Mary is short” and the Spanish sentence “María es alta” and tries to relate them by translation. This task is frustrated, because “María es alta” translates to “Mary is tall.” How can one and the same Mary/María be both short and tall? Perhaps Mary/María is a person who lacks any objective height? This proposal is analogous to the majority view about gravitational energy: one builds the pseudotensor using the same functional form with the metric tensor components related using the tensor transformation law and finds the pseudotensor ascribing rival gravitational energy properties to the same points relative to different coordinate systems. Or perhaps there is no Mary/María, because incompatible properties are ascribed to this putative woman? When Mary and María walk into the room together, the difficulty vanishes: $Mary \ne Mar\acute{\mathrm{\i }}a$, so why should two different people have the same properties? Instead of a description of one person in two languages, one had a description of two different people, each in her own native language. Languages, like coordinate systems, are arbitrary conventional ways to express realities that transcend those specific conventions; translation is like the transformation rule for a tensor or other geometric object. Much as people have native languages (typically unique), conserved energies have native coordinate systems, such as those in which the corresponding translation vector takes the form $\xi ^{\mu } =(1,0,0,0),$ which allows one to pull the vector components out of the natural conserved current equation $\partial _{\mu } (\xi ^{\nu } {\mathcal {T}}^{\mu }_{\nu })=0$ [or some generalization thereof (Sorkin 1977)] to get a more coordinate-dependent equation for a conserved rank **2** expression $\xi ^{\nu } \partial _{\mu } {\mathcal {T}}^{\mu }_{\nu }=0$ (all indices being in their primordial locations).^8^ The conventional tacit assumption in General Relativity has been that there is only one energy (or four energy-momenta) for a gravitational energy-momentum pseudotensor to describe; thus the one energy (or four energy-momenta, rather) must be described in all coordinate systems, albeit with frustratingly incompatible properties. But the assumption that there is (at most) only one energy was traditionally motivated largely by lack of imagination (Pitts 2009b, 2010); it is difficult to find anyone entertaining the idea that one could be a realist about the mathematics with infinitely many conservation laws.^9^ One can now reject the idea out of a wish for simple bookkeeping or preference for ontological parsimony, perhaps, though whether it is parsimonious to accept material energy and reject gravitational energy when the two have so much in common in their formal origins, justification and participation in a conservation law is unclear. If one uses Noether-inspired bookkeeping, one would expect infinitely symmetries to yield infinitely many momenta. If a pseudotensor describes infinitely many energies, each with its own preferred coordinate system, then pseudotensorial behavior becomes intelligible.

Indeed one can go further: pseudotensorial behavior becomes inevitable and virtuous, because a geometric object with finitely many components (presumably 10 or 16) could only describe one energy, its components in each coordinate system all being merely faces of one and the same conserved quantity. Pseudotensoriality permits something that would be impossible otherwise: it enables a finite-component object to describe infinitely many conserved quantities, which one expects to exist due to the infinity of symmetries. Hence pseudotensorial behavior is a virtue, not a vice, from this perspective. A publisher who prints Jane Austen in English, Goethe in German, and Dostoyevsky in Russian will not use nearly so much paper as one that publishes (translations of) every book in every language (the analog of tensor calculus). The publisher printing only works in their original languages admittedly makes more demands on the readership. But the policy is not unimaginable or absurd. Or consider translations of the claim that “the flag is red, white and blue.” This claim works well in most predominantly English-speaking countries and well enough in France, but goes badly in Germany, Spain, and most other countries. The reason is not far to seek: the most obvious referent for the flag (*bandera*) in Spanish is the flag of Spain or some other Spanish-speaking country, not the flag of America, the United Kingdom, Australia or New Zealand. Marshall McLuhan wrote that “the medium is the message.” When it comes to gravitational energy-momentum pseudotensors, publishers, and flags, the mediating conventions might well be strongly correlated with the message.

If one takes these conservation laws seriously, then the situation for General Relativity, one might think, is basically the same as for other field theories; the dualist still can (and must) appeal to the (bi)conditional nature of conservation laws to deflect the objection. (Actually one needs to work harder than that, as will appear below!) People who find it absurd that General Relativity should be a resource for dualists, as I do—in my experience physicists tend to recoil from Collins’s inference when confronted with it (Pitts 2009b)—could take that judgment as a motivation to take gravitational energy-momentum pseudotensor conservation laws more seriously. Doing so would also perhaps leave more room for conserved quantity theories of causation, as will be discussed again at the end.

What makes the energies all energies? There seem to be four components. First, they participate in the conservation laws in the usual way determined by symmetries of the Lagrangian. Second, they are determined as functions of the physical fields and their spatio-temporal rates of change by a standard algorithm that works all Lagrangian-based classical field theories. Third, the vector fields describing the symmetries are time-like, so the conserved quantities are energies rather than momenta or angular momenta or the like. Fourth, in familiar special cases they mathematically approximate or overlap with more familiar energies from pre-general relativistic theories. That is all just as in other theories [except that not only the vector describing the symmetry of the action, but a whole basis is needed, except using Komar’s description (Komar 1959), which has its own difficulties (Katz 1985)]. For technical reasons there are a great many more of energies than in earlier theories.

While such ideas strike me as illuminating, the reader does not need to sympathize with them in order to profit from the covariant calculations below using the generalized Bianchi identities. Those calculations, however, reflect some plausibility back onto the realism about pseudotensorial energy that had the correct heuristic force.

## Bianchi Identities Constrain Mental Influence

A more clearly compelling reason that General Relativity does not help the dualist is that one can show mathematically that whereas pre-GR theories will tolerate the introduction of an external mental potential(s) without objection [with symmetries and conservation laws both spoiled thereby in accord with Noether’s first theorem and converse (Noether 1918)], General Relativity *actively resists* such an external influence by striving to make it vanish. One can, but need not, construe this point as a general relativistic energy conservation objection. Carroll’s undefended claim that nonphysical mental causation would “overthrow everything we think we have learned about modern physics” (Carroll 2011) cannot be defended (at least by him) in terms of energy conservation because he denies it (Carroll 2010); his General Relativity textbook does not apply Noether’s theorem to General Relativity (Carroll 2004). However, his clearly framing the question of how to modify the Dirac-Maxwell-Einstein equations to permit immaterial mental influence suggests a research question. If one attempts to answer this question, then a new difficulty for interactionist dualism from the generalized Bianchi identities emerges. If one introduces souls that act on physical fields in theories different from General Relativity, then some of the conservation equations will be false,^10^ because the souls act as sources/sinks for energy and momentum. But in General Relativity, the generalized Bianchi identities, related to Noether’s *second* theorem, are also relevant. In this application one can show that they tend to constrain how the soul could act on matter. General Relativity presents dualism not a new solution as Mohrhoff and Collins envisaged, but a new problem of surprising technical intricacy.

General Relativity proves very different from earlier physical theories in that whereas earlier theories passively tolerate the introduction of external causal influences (applied fields, external potentials), General Relativity actively resists them by trying to force them to vanish. One might take the view that laws of nature describe the tendencies of physical entities, while leaving unspecified whether any nonphysical entities influence the physical (von Wachter 2006). The Bianchi identities exhibit how and to what extent resistance occurs.

Let the action for General Relativity with matter *u* (indices suppressed) and influence $\Psi$ from immaterial minds be $S[g_{\mu \nu },u, \Psi ]$. While little can be said with any confidence about how the mental influence $\Psi$ enters in, let us treat it as a field (or collection thereof) with some definite coordinate transformation properties, entering the action algebraically or with up to some finite number of derivatives, with $S[g_{\mu \nu },u, \Psi ]$ invariant (at least up to a boundary term) under coordinate transformations as usual.^11^ Crucially, $\Psi$ does *not* have any equations of motion from the principle of least action: one does not postulate the vanishing of $\frac{ \delta S}{\delta \Psi }$. (One might assume the boundary condition that $\Psi =0$ outside brains, or wherever immaterial minds might act.) Using coordinate transformations that are trivial at the boundary (to annihilate boundary terms), one has

1 $$\begin{aligned} \int d^4x \left( \frac{ \delta S}{\delta g_{\mu \nu }} \pounds _{\xi } g_{\mu \nu } + \frac{ \delta S}{\delta u} \pounds _{\xi } u + \frac{ \delta S}{\delta \Psi } \pounds _{\xi } \Psi \right) =0. \end{aligned}$$

Because of the matter field equations $\frac{ \delta S}{\delta u} = 0$, the second term vanishes. Because of the gravitational field equations $\frac{ \delta S}{\delta g_{\mu \nu }}=0$ (Einstein’s equations or some modification thereof), the first term vanishes.

To go further, it is necessary to be a bit more specific about the mental influence $\Psi .$ To emphasize the possibility of multiple components, one can write $\Psi$ as $\Psi ^A,$ where the index *A* runs over all the components. Suppose (with little loss of generality) that under infinitesimal coordinate transformations along the vector field $\xi ^{\mu },$ $\Psi ^A$ transforms as

$$\begin{aligned} \pounds _{\xi } \Psi ^A = \xi ^{\mu } \partial _{\mu } \Psi ^A - C^{A\nu }_{\mu }(\Psi , g_{\rho \sigma }?) \partial _{\nu } \xi ^{\mu } \end{aligned}$$

(Anderson 1958). This expression lacks a term independent of $\Psi ^A$ and so suits the assumption above that $\Psi ^A=0$ is physically distinguished as the state when the soul does nothing to the body. While $C^{A\nu }_{\mu }(\Psi , g_{\rho \sigma }?)$ is presumably linear in $\Psi ^A$, allowing the transformation rule for $\Psi$ to depend also on $g_{\rho \sigma }$ leaves room for $\Psi ^A$ to contain spinor fields if desired.^12^

The terms that survive when the gravitational and material field equations are imposed takes the form

2 $$\begin{aligned} \int d^4x \frac{ \delta S_m}{\delta \Psi ^A} \left[ \xi ^{\mu } \partial _{\mu } \Psi ^A - C^{A\nu }_{\mu }(\Psi , g_{\rho \sigma }?) \partial _{\nu } \xi ^{\mu }\right] =0. \end{aligned}$$

Integrating by parts, discarding boundary terms because the coordinate transformations are assumed to be trivial at the boundary, and using the arbitrariness of $\xi ^{\mu }$ in the interior to remove the integration implies

3 $$\begin{aligned} \frac{ \delta S}{\delta \Psi ^A} \partial _{\mu } \Psi ^A + \partial _{\nu } \left[ C^{A\nu }_{\mu }(\Psi , g_{\rho \sigma }?) \frac{ \delta S}{\delta \Psi ^A} \right] =0. \end{aligned}$$

Now let us make the simplest assumption, that the mental influence is described by a scalar field (just one function, the same in all coordinate systems; a pseudoscalar, changing sign under negative-determinant coordinate transformations, would work the same way). In this case $C^{A\nu }_{\mu }(\Psi , g_{\rho \sigma }?) =0.$ One can consider various possibilities for $\frac{ \delta S}{\delta \Psi }$; with only one component, $\Psi$ does not need the index *A*. Then the Lie derivative $\pounds _{\xi } \Psi$ along the vector field $\xi ^{\mu }$ is just the directional derivative. The equation above becomes

4 $$\begin{aligned} \frac{ \delta S}{\delta \Psi } \xi ^{\mu } \partial _{\mu } \Psi =0. \end{aligned}$$

Because $\xi ^{\mu }$ is arbitrary due to general covariance, one therefore has

5 $$\begin{aligned} \frac{ \delta S}{\delta \Psi } \partial _{\mu } \Psi =0. \end{aligned}$$

If $\frac{ \delta S}{\delta \Psi } \equiv 0$ (because $\Psi$ appears only in a boundary term), then $\Psi$ plays no role and can be eliminated from the discussion. Another possibility (which appears in a toy example below) is that $\frac{ \delta S}{\delta \Psi } \equiv constant\ne 0$; then spatiotemporal constancy of $\Psi$ follows immediately. Assuming then that $\frac{ \delta S}{\delta \Psi }$ is not identically a constant (0 or not), suppose that $\frac{ \delta S}{\delta \Psi }$ does not depend on $\Psi$ but depends on $g_{\mu \nu }$ and/or *u*. Then wherever $\Psi$ varies spatiotemporally ($\partial _{\mu } \Psi \ne 0$), as it must to have any chance of representing the mind’s action on the body, the resulting equation  will impose a novel restriction on the allowed states of matter and gravity like a Lagrange multiplier even without the *postulation* that $\frac{ \delta S}{\delta \Psi }=0$. (A nonzero constant $\Psi$ everywhere and always is not suitable for representing the influence of spatiotemporally localized minds, so if $\partial _{\mu } \Psi =0,$ the boundary condition $\Psi =0$ implies that $\Psi$ vanishes throughout space-time.) Hence $\Psi$ either does nothing or unreasonably restricts the physical possibilities for gravity and matter, or perhaps does nothing in some parts of space-time and unreasonably restricts the physical possibilities for gravity and matter elsewhere; clearly this option fails. Another possibility, which one might write loosely as  , is that $\frac{ \delta S}{\delta \Psi }$ depends on $\Psi$ algebraically but not on derivatives of $\Psi .$ [In this case $\Psi$ is almost an auxiliary field, where an auxiliary field has Euler–Lagrange equations in which it appears algebraically and so can be solved for (Pons 2010).] In order that $\Psi$ not interfere with the dynamics of gravity and matter where and when $\Psi$ is ‘turned off’ (such as outside brains), the algebraic-in-$\Psi$ equation  must have as a solution $\Psi =0.$ While there might be other solutions as well—a cubic equation might have 3 real solutions, *etc.*—presumably they will be countable and will not fill an interval including $\Psi =0.$ It seems impossible that $\Psi$ jump from 0 to some nonzero solution of  . If $\Psi$ cannot slide continuously from 0 to small nonzero values while satisfying the relevant algebraic equation, then this algebraic-in-$\Psi$ option does not work, either. The remaining possibility (at least if non-local coupling is excluded) is that $\frac{ \delta S}{\delta \Psi }(g_{\mu \nu }, u, \Psi ) = 0$ depends on $\partial _{\mu }\Psi ;$ it might or might not depend algebraically on $\Psi$ as well.

An example might help: let $\Psi$ appear in the Lagrangian density *via* the expression $-\frac{1}{2} \sqrt{-g} g^{\mu \nu } (\partial _{\mu }\Psi ) \partial _{\nu } \Psi ,$ like a massless scalar field. Then the equation $\frac{ \delta S}{\delta \Psi } \partial _{\mu } \Psi =0$ implies that at any space-time point either $\Psi$ is spatio-temporally constant or it satisfies the wave equation $\partial _{\mu } (\sqrt{-g} g^{\mu \nu } \partial _{\nu } \Psi ) =0.$ While most solutions of the wave equation do vary spatiotemporally (a step in the right direction), they are also fixed by initial and boundary data. Prior to the existence of minds acting on bodies, $\Psi$ and its time derivative should vanish, yielding $\Psi =0$ everywhere and always due to the wave equation. Hence this partial differential equation is still too strong to leave $\Psi$ the wiggle room needed to represent the soul’s action on the body. Assuming that the wave equation example is representative, it seems that local classical field theory provides no options suitable for modelling the effect of the mind on the body if such effects are mediated by a single scalar (or pseudoscalar) function.^13^ Hence either there is no such dualist mental causation after all, or its influence is not represented by one scalar (or pseudoscalar) field, or some other Lagrangian makes a relevant qualitative difference. That result contrasts sharply with what would happen in a special relativistic field theory, namely, that the energy-momentum currents would fail to be conserved where and when the mental causation occurs, and the theory does not try to force would-be mental causation to disappear. Regardless of one’s interpretation of gravitational energy and general relativistic conservation laws, General Relativity makes mental causation more difficult by excluding at least the simplest case.

What if there are two, three or four scalar fields? If their gradients are linearly independent, then one will have two, three or four copies (respectively) of the same sad story. One can use the scalars with independent gradients as space-time coordinates locally and thus turn the $\partial _{\mu } \Psi ^A$ expressions into a rectangular matrix with two, three or four entries with the value 1 and the rest 0. But if the scalars’ gradients are not all linearly independent, then one has correspondingly fewer equations and correspondingly fewer unknowns. So evidently even up to four scalar fields cannot do the job; success, if possible at all, would require more scalars (at least 5), non-scalars, or some combination of the two.

Withdrawing the simple assumption that $\Psi$ is a scalar field and allowing it to be a quite arbitrary collection of fields $\Psi ^A,$ one has the more general relation

$$\begin{aligned} \frac{ \delta S}{\delta \Psi ^A} \partial _{\mu } \Psi ^A + \partial _{\nu } \left[ C^{A\nu }_{\mu }(\Psi , g_{\rho \sigma }?) \frac{ \delta S}{\delta \Psi ^A} \right] =0. \end{aligned}$$

from above. The question arises whether these relations are still strong enough to annihilate the soul’s influence. Note that there are *still only four of these identities*,^14^ but there is *no limit* on how many field components over which the index *A* can range. The freedom to choose the form of the transformations of the fields is also relevant, albeit in a less transparent and less important way. In the single scalar $\Psi$ case, the generalized Bianchi identities exclude the soul’s influence, which is forced to constancy and then vanishing everywhere. But if $\Psi ^A$ has enough components (5 or perhaps a bit higher being a plausible first estimate), then arguably the soul ‘wins’ and manages to act as it wishes even in a general relativistic world. Perhaps the soul deploys four scalar fields sacrificially as pawns to saturate the generalized Bianchi identities while the more commanding field component(s) undertake to implement the soul’s intentions in the world. If there are, for example, 5 scalar fields in $\Psi ^A,$ let $\Psi ^4$ try to impose the soul’s intentions on the world (or at any rate on the brain). Can it get by with a little help from its friends, the other four scalar fields? It seems plausible that one (call it $\Psi ^0$) could accommodate dependence on coordinate $x^0,$ another (call it $\Psi ^1$) could accommodate dependence on $x^1,$ another (call it $\Psi ^2$) on $x^2,$ and another (call it $\Psi ^3$) on $x^3,$ locally, for example, so as to satisfy the generalized Bianchi identities without forcing $\frac{ \delta S}{\delta \Psi ^4} \partial _{\mu } \Psi ^4 =0$. That last equation would seem to block the soul’s effort. [This would not be the first time in General Relativity that one needs to invest 5 scalar fields to keep 1 of them (Bergmann 1962, pp. 253, 254; Rovelli 2004, pp. 81, 82).] This function-counting argument is admittedly crude and merely plausible. A more definitive resolution would require a more specific postulate about how many field components of various types $\Psi ^A$ contains and how they couple to the metric $g_{\mu \nu }$ and matter fields *u*. Thus it could turn out that the general relativistic objection still has force even with some additional field components to employ. But that seems unlikely to be true to any great extent. There is a literature on underdetermined differential equations, which in general outline confirms the expectation that having fewer equations than unknowns leads to having a variety of solutions (Bender et al. 2000; Elkin 2009).

*Mutatis mutandis* a similar discussion would apply to (special) divine action (a.k.a. miracles), angelic action, demonic action, genie action (genies/jinn being part of Muslim belief), action by the spirits presumably of the dead [which was accepted by a number of distinguished Victorian scientists who studied the matter in séances and the like (Wallace 1905)], or action of whatever wholly or partly immaterial personal beings someone might wish to consider. Note that the description above does *not* supply a field $\Psi$ for *each* spirit. If there can be multiple angels in the same place [a topic of renewed interest recently in analytic philosophy (Hawthorne and Uzquiano 2011)], then one could consider how to describe such scenarios. But the discussion has strayed near, if not past, the boundaries of contemporary plausibility structures already.

Some readers might share Carroll’s experience: “[n]obody ever asks these questions out loud, possibly because of how silly they sound.” While I don’t altogether disagree, in my estimation the asking and answering of these questions pays for itself, if in no other way, by the fruit yielded regarding gravitational energy in General Relativity and possibly also in the consequences for conserved quantity theories of causation. One might also respond that the questions have a sort of timeless interest; a few centuries centuries ago such questions, or at any rate close analogs of such questions, were very seriously debated by leading thinkers (Watkins 1995, 1998), whereas some current questions (such as how consciousness is possible for a purely physical system) not only sounded silly to many people, but attracted strong arguments in refutation, such as Leibniz’s mill argument:

Furthermore, one is obliged to admit that *perception* and what depends upon it is *inexplicable on mechanical principles*, that is, by figure and motions. In imagining that there is a machine whose construction would enable it to think, to sense, and to have perception, one could conceive it enlarged while retaining the same proportions, so that one could enter into it, just like into a windmill. Supposing this, one should, when visiting within it, find only parts pushing one another, and never anything by which to explain a perception. Thus it is in the simple substance, an not in the composite or in the machine, that one must look for perception (Rescher 1991, p. 83).

This kind of argument has contemporary proponents (Plantinga 2007; Ruse 2018). Without endorsing the argument, Markus Gabriel calls it “one of the earliest modern formulations of the so-called **hard problem of consciousness**” (Gabriel 2017, p. 121, emphasis in the original). The de-materialization of matter into fields with basically just mathematical properties seems not to affect the force of the argument. Whether one struggles to take seriously a mathematical description of the mind-body problem in General Relativity due to materialist sympathies or not, it is sometimes useful to step out of one’s view and see to what extent the weight of argument forces one back into it. The analogy of destructive testing, understanding something by breaking it, comes to mind. One non-dualist undertook such an exercise and concluded that the arguments against dualism weren’t very compelling (Lycan 2009). Here I have taken Carroll’s foundling research questions (or analogs suited to classical field theory) seriously enough to do some calculations and find some results that, in some cases, are very congenial to Carroll’s views, hence providing an argument rather than an assertion.

### More Options from Inequivalence Under Field Redefinitions

Another surprise is that there are more *distinct* ways that the distinctively mental influence $\Psi$ on matter might be realized than one might have expected, because some options that are equivalent in more standard physical contexts become distinct in the present problem. Suppose that one is trying to write down a toy mathematical description of how the distinctively mental influence (the soul’s effect) relates to gravity and matter. The interaction term coupling the soul to gravity and matter should respect general covariance and thus be a scalar under coordinate transformations. Hence the integrand should be a scalar density of weight 1. Ignoring matter and letting the soul couple to gravity only for simplicity (to make an important point vivid), two obvious choices (out of an uncountable infinity) are:

$$\begin{aligned} \int d^4x \sqrt{-g}(x) \Psi (x) \end{aligned}$$

(the soul’s influence $\Psi$ acts as a scalar field, the same in all coordinate systems), or

$$\begin{aligned} \int d^4x {\tilde{\Psi }}(x) \end{aligned}$$

(the soul’s influence acts as a scalar density of weight 1). Usually in physics one regards such choices as equivalent because one can relate the two choices by ${\tilde{\Psi }} = \sqrt{-g} \Psi .$ In cases where all fields satisfy the principle of least action, it is easy to find that the resulting equations of motion are unaffected by field redefinitions. An analogous result holds in quantum field theory (Duff 1981). But now suppose (as holds in our case) that some fields satisfy the principle of least action and some do not. A typical application is electromagnetism in special relativity with arbitrary coordinates; the flat metric tensor of Special Relativity appears in a mildly non-trivial form because one can label the flat space-time in an arbitrary way (coordinates can be angles or can slosh around because your body or the Earth or the Sun defines the origin, or whatever). A variation of the action *S* made by varying the fields $A_{\mu }$ and $\eta ^{\mu \nu }$ (the inverse of $\eta _{\mu \nu }$), with the variations being 0 at the boundary, is

6 $$\begin{aligned} \delta S= \int d^4x \left( \frac{\delta S}{\delta A_{\mu }}(x) \cdot \delta A_{\mu }(x) + \frac{\delta S}{\delta \eta ^{\mu \nu } }|A \cdot \delta \eta ^{\mu \nu }(x) \right) , \end{aligned}$$

where |*A* indicates the holding constant of $A_{\mu }.$ The Euler–Lagrange equations for electromagnetism are $\frac{\delta S}{\delta A_{\mu }}(x)=0.$ Now let us introduce a new field $B^{\nu } = A_{\mu } \eta ^{\mu \nu }.$ One can use $B^{\nu }$ and $\eta ^{\mu \nu }$ as a new set of fields (so that $B^{\nu }$ is now primitive and the relation $B^{\nu } = A_{\mu } \eta ^{\mu \nu }$ now defines $A_{\mu }$) and vary the action:

7 $$\begin{aligned} \delta S= \int d^4x \left( \frac{\delta S}{\delta B^{\nu }}(x) \cdot \delta B^{\nu }(x) + \frac{\delta S}{\delta \eta ^{\mu \nu } }|B^{\nu } \cdot \delta \eta ^{\mu \nu }(x) \right) , \end{aligned}$$

with Euler–Lagrange equations $\frac{\delta S}{\delta B^{\nu }}(x)=0$. The relation $B^{\nu } = A_{\mu } \eta ^{\mu \nu }$ lets one write $\delta B^{\nu } = A_{\mu } \delta \eta ^{\mu \nu } + \eta ^{\mu \nu } \delta A_{\mu }.$ Equating coefficients of $A_{\mu }$ and of $\delta \eta ^{\mu \nu }$ yields, respectively,

8 $$\begin{aligned}&\frac{ \delta S}{\delta A_{\mu } } = \frac{\delta S}{\delta B^{\nu } } \eta ^{\mu \nu }, \end{aligned}$$

9 $$\begin{aligned}&\frac{\delta S}{\delta \eta ^{\mu \nu } }|A = \frac{\delta S}{\delta \eta ^{\mu \nu } }|B + \frac{\delta S}{\delta B^{(\nu } } A_{\mu )} \end{aligned}$$

(where the parentheses imply symmetrization). One finds that the electromagnetic field equations are equivalent, and the (Hilbert–Rosenfeld) stress-energy tensor (basically $\frac{\delta S}{\delta \eta ^{\mu \nu }}$) changes only by terms that are 0 when the electromagnetic field equations hold (Pons 2011; Pitts 2016c). In this case one has redefined the dynamical field by folding some non-dynamical field into it ($B^{\nu } = \eta ^{\mu \nu } A_{\mu }$), but the non-dynamical inverse flat metric tensor $\eta ^{\mu \nu }$ is left alone. Hence it doesn’t matter if electromagnetism is a covariant vector $A_{\mu }$ or a contravariant vector $B^{\nu }.$ The usual covariant choice has the virtue of not requiring the construction of covariant derivatives and so perhaps is preferable.

But the case of the soul’s influence on gravity (and other physical fields) is different, because the entity not redefined, gravity, has Euler–Lagrange equations but the redefined entity does not. By an analogous derivation one finds that new and old Euler–Lagrange equations differ. Does the soul’s influence transform as (e.g.) a scalar or a scalar density of weight 1? (It should be possible to describe the soul’s influence in any admissible coordinate system within General Relativity, so questions of this sort have to make sense, however incongruous they might sound at first.) Now the dynamical field $\sqrt{-g}$ (which defines volumes in General Relativity), for which the principle of least action applies (because it is part of the space-time metric $g_{\mu \nu }$), is left alone, while the mental influence $\Psi ,$ which does *not* satisfy the principle of least action, is redefined by folding in some of $\sqrt{-g}.$ Using the generalized Bianchi identities as above, one can easily find that the term $\int d^4x \sqrt{-g}(x) \Psi (x)$ (the soul acting as a scalar) leads to a cosmological constant ($\Psi =constant$), altering the gravitational field equations in the same way throughout the whole history of the universe, which is startling for an influence that was supposed to be confined to my brain (and my lifetime)—unless one imposes the boundary condition $\Psi =0$ at the boundaries, in which case $\Psi =0$ everywhere and always, so the soul does nothing at all. The term $\int d^4x {\tilde{\Psi }}(x)$ (the soul’s influence as a scalar density of weight 1), by contrast, definitely does nothing at all (even without appeal to boundary conditions) because it does not couple to anything physical; that is also disappointing, but in a *different* way. Not appealing to boundary conditions in one case and needing boundary conditions in another shows that the two cases are inequivalent.

The point is not that two ludicrously oversimplified examples behave badly, but that they are *inequivalent*, one not needing boundary conditions and the other needing them—contrary to what one might have assumed based on related but subtly different experiences with field redefinitions in fundamental physics. The two cases’ Euler–Lagrange equations for gravity (the Einstein-like equations) differ. Explicitly one has for the variational derivatives

10 $$\begin{aligned}&0 {\mathop {=}\limits ^{?}} \frac{\delta S}{\delta \sqrt{-g} }|\Psi = \frac{\delta S}{\delta \sqrt{-g} }|{\tilde{\Psi }} +\frac{\delta S}{\delta {\tilde{\Psi }} } \frac{{\tilde{\Psi }}}{\sqrt{-g}} {\mathop {=}\limits ^{?}} 0 , \end{aligned}$$

11 $$\begin{aligned}&\frac{\delta S}{\delta \Psi } = \frac{\delta S}{\delta {\tilde{\Psi }}} \sqrt{-g} \ne 0. \end{aligned}$$

The first equation, which shows $10\%$ of Einstein’s equations (the trace, that is, the sum of the diagonal components of the equations) in the presence of mental causation, shows that the gravitational field equations differ by a term involving the soul’s influence and thus are not equivalent. Whether the soul’s influence is a scalar, or a scalar density of weight 1, or something(s) else, makes a difference in the field equations. One might have expected $\frac{\delta S}{\delta \sqrt{-g} }|\Psi = 0$ to be equivalent to $\frac{\delta S}{\delta \sqrt{-g} }|{\tilde{\Psi }} =0,$ but one sees that in fact they differ by a nonzero term $\frac{\delta S}{\delta {\tilde{\Psi }} } \frac{{\tilde{\Psi }}}{\sqrt{-g}}.$ Admittedly, the generalized Bianchi identities substantially reduce this difference by forcing the soul’s influence (rescaled by a suitable power of $\sqrt{-g}$) to be constant, but do not go so far as to establish equivalence of the field equations, which differ by a cosmological constant. (Imposing boundary conditions to make that constant influence 0 does yield equivalence, but that is too weak: using boundary conditions is inequivalent to not using them.) Analogously, one needs to contemplate in principle distinct vector-like possibilities: a covariant vector, a contravariant vector, a covariant vector density of any real weight, and a contravariant vector density of any real weight are not one option, but $2\infty$ that might differ. Obviously there are other possibilities: various flavors of spinor fields, *etc.*, not to mention multiple scalars, multiple scalar densities, a scalar and vector of some sort, *etc.* Thus not only can $\Psi ^A$ involve any kinds of fields in whatever quantities you like, but also some cases that one might have expected to be equivalent, in fact differ. Hence there is in principle an enormous zoo of possibilities that could be explored if one aimed (as I do not) to try to give some positive realistic account of how immaterial minds might act in a general relativistic physical world. If one wishes to supply Carroll’s missing argument, then one has a great many distinct options to consider. If some options survives this elimination process, the result will still be very far from biological realism, presumably. But that is a different kind of argument, one best left to neuroscientists rather than theoretical physicists and philosophers of physics.

## Whom Does the General Relativistic Objection Affect?

The new general relativistic objection from the Bianchi identities (perhaps one can say, from energy conservation) is not obviously question-begging even given Noether’s first theorem and its converse. Thus it is a better argument than the traditional Leibnizian one. But how good an objection is it? Against which kinds of interactionist substance dualists (or non-epiphenomenalist property dualists) is it effective?

As noted above, the power of the Bianchi identities to constraint the mental influence $\Psi ^A$ depends especially on the number and to some extent on the type of fields included in $\Psi ^A$. General Relativity implies (in 4 space-time dimensions) 4 Bianchi identities. General Relativity thus puts up a nonzero but bounded amount of resistance to external forces, unlike earlier theories that put up no resistance at all. But what determines how many components there are in $\Psi$? The answer, surely, depends on what kind of process produced human beings.

If it was a purely *naturalistic* evolutionary process, then any immaterial self must evolve naturally. Such is, one might think, impossible. If so, then dualism is already ruled out without the need to appeal to the Bianchi identities to constrain interactionism. If perchance an immaterial self could evolve, perhaps evolving components of $\Psi ^A$ is difficult and hence improbable; maybe the physicists’ exponentially suppressed probability from statistical mechanics is relevant? Then $\Psi ^A$ should have few components; perhaps 5, roughly the minimum number needed to overwhelm the Bianchi identities, is too many? (It is difficult to speak sensibly about this question; perhaps one should remain silent.) Or if it is both possible and not difficult to evolve $\Psi ^A,$ it will still be random (apart from natural selection, which is not the productive phase of evolution), not engineered so as to give $\Psi ^A$ enough components to overwhelm the Bianchi identities and let the soul act on the world. So it would seem that if souls somehow manage to evolve naturally and acquire field components $\Psi ^A$ with which to try to act on the physical world, then there is still a reasonable chance that the Bianchi identities will drown $\Psi ^A$, forcing it to be 0. The prospects for interactionism given a purely naturalistic evolutionary process do not seem all that bright.

And yet such a conclusion seems to conflict with the views of some interesting people. If one attempts to look for nontheists (for whom *naturalistic* evolution will be required) who defended or at least sympathized somewhat with substance dualism or allied doctrines about spirits, one does not come up empty in the twentieth century. Idealist McTaggart (Nathan 1991) is perhaps appropriately listed, though whether his spirits acted on matter would require care. Broad was doubtful about a personal God (Broad 1953), sympathetic to parapsychology (Broad 1937, p. 395, 1953), and unwilling to criticize substance dualism on grounds of energy conservation (Broad 1937, pp. 106–108) [though his response to that argument was fallacious (Pitts 2019)]. Turing took the statistical evidence for telepathy to be overwhelming (Turing 1950). More recent figures include Popper (Popper and Eccles 1983) and parapsychology expert John Beloff (Beloff 1962; Smythies and Beloff 1989). Beloff intended to be surprised if he still existed after death (though not annoyed like Broad!) (Steinkamp 2002, p. 13) and was an atheist (Braude 2006; Blackmore 2006). Some more recent authors also aim to incorporate spiritual/parapsychological phenomena or entities within a broadened naturalist rather than supernaturalist framework (Griffin 1997; Grosso 2016). Against people who affirm causally active spirits but not God, the general relativistic objection may have some force. This category should perhaps also contain people influenced by nontheistic Buddhism. Perhaps at least some who work on evidence for reincarnation [a topic on which Carl Sagan had respectful words (Sagan 1979, pp. 47, 48)] [e.g., (Stevenson 1983, 1997; Pasricha 1990, 2008)] would also qualify, though I cannot comment on Stevenson’s or Pasricha’s views. Thus one should not exaggerate the connection between dualism and theistic belief, because parapsychologists who take themselves to be doing science frequently are not theists.

Because the strength of the Bianchi identity-based objection to Cartesian mental causation diminishes, the more fields are at the soul’s disposal, it follows that the seriousness of the objection winds up being interestingly related to the theism-atheism debate. Without God, a soul’s acquiring fields of influence on the physical world is presumably impossible, or difficult, or random, and hence has a reasonable chance of not overcoming the tendency of the Bianchi identities to trivialize the mental influence. But given theism of a traditional stripe (perhaps a form of theistic evolution), a soul’s possessing such fields appears to be possible, not difficult, and designed for the task at hand, hence highly likely to succeed in overcoming the Bianchi identities to let the soul act on the physical world. Perhaps the import of the new general relativistic objection, then, is that *atheists shouldn’t be interactionist dualists*. Of course not many atheists have been interactionist dualists anyway, at least not in the West, largely due to arguments not involving conservation laws [though energy conservation did play a substantial role in moving people away from dualism (Wegener 2009)]. There are prominent examples from the recent past, however, as noted above. The new result from General Relativity might put pressure on views that affirm souls but do not affirm God (such as some forms of Buddhism and perhaps forms of spiritualism) or that claim to incorporate spiritual/parapsychological phenomena or entities within a broadened naturalist rather than supernaturalist framework (e.g., Broad 1953; Griffin 1997; Cardeña et al. 2015; Grosso 2016). But at least for theistic views (whether Abrahamic, Hindu or some other kind) and naturalism, the new general relativistic objection does not ultimately make much difference for most people: typically either the objection doesn’t work, or its conclusion was already accepted anyway on other (comparably good) grounds. In that sense, the new general relativistic energy conservation objection and the old Leibnizian energy conservation objection are somewhat alike: neither works very well in motivating many people to change their beliefs, though the general relativistic objection is better because it does discourage some views, namely those involving causally active souls without God.

On the other hand, it is noted by people of various views that there is a certain natural harmony between dualism and theism (Bain 1873, chapter 7; Taliaferro 1994; McGinn 1999; Foster 2001; Wiebe 2004; Plantinga 2007; Moreland and Rae 2000, p. 352; Moreland and Craig 2003, pp. 262–265). Thus objections to interactionism that presuppose nontheism will not be all that effective against this large class of substance dualists. Supposing that God exists and (e.g.) somehow guided evolution, presumably there are enough of and the right kinds of $\Psi ^A$ components to overcome the restriction imposed by the Bianchi identities. Perhaps there is an imperfect analogy to this remark: “Nature may shout no, but human ingenuity...may always be able to shout louder.” (Lakatos 1971) Some examples from the eighteenth century German debate on Leibniz’s objection and pre-established harmony are useful. Johann Peter Reusch, a Wolffian, rejected pre-established harmony partly on the grounds that it made bodies superfluous. He concluded that there is no sufficient reason for creating bodies (Watkins 1998), a serious objection for a Wolffian. Likewise Christian August Crusius rejected the conservation laws,

noting that if they were true, the absurd results would follow that minds could not cause any motion and, as Reusch had noted earlier, that matter would not be able to fulfill the purpose for which God intended them [*sic*], namely to be a means for rational and free beings (Watkins 1998).

The attached footnote translates Crusius: “But then the material world would be of no use to minds, and [it] would have been created completely without a purpose” (Watkins 1998, footnote 164; 1995, footnote 98). To be sure, nowadays there exist Christians who take physicalist views about human persons (van Inwagen 1995; Baker 1995; Murphy 1998; Merricks 1999; Corcoran 2006) and so might be able to evade Crusius’s argument.

Perhaps the import of the development of Carroll’s foundling is that *nontheists* have an additional reason to deny causally efficacious souls. This point isn’t entirely uninteresting because it undermines a class of views that has in fact been and still is held in some circles. [That result contrasts with Leibniz’s objection, which fails entirely even at the classical level, making appeal to quantum mechanics unnecessary (Pitts 2019).] In any case General Relativity does not make mental causation easier, but can make it harder in some contexts. Nontheistic dualists therefore might need to hope for the truth of the sometimes-heard claim that quantum mechanics facilitates soul-to-body causation.

## Philosophical Payoff Outside the Philosophy of Mind

While one might think that the relevance of relating General Relativity to the traditional mind-body problem and especially the Leibnizian energy conservation strand would not propagate beyond the philosophy of mind and associated sectors of metaphysics, arguably there is in fact a payoff in two other areas, one in the foundations of physics and one in philosophical theories of causation, as hinted earlier.

### Bearing on Gravitational Energy (Anti)Realism and Conservation

The treatment of the generalized Bianchi identities above shows that the relevance of General Relativity for mental causation does not have to be yoked to the frequently denigrated pseudotensor conservation laws for energy and momentum. Rather, the tendency of General Relativity to restrict immaterial-to-material causation can be explored in a tensorial way, free of conventional taint and hence free of the usual interpretive controversy over gravitational energy. The fact that these calculations do show a tendency to resist immaterial-to-material causation shows that the heuristic force of the widely received anti-realist gloss on the conservation laws is incorrect. That heuristic was the basis for the Mohrhoff–Collins view that General Relativity facilitates immaterial-to-material causation. The conclusion that General Relativity makes immaterial-to-material causation harder rather than easier could have been motivated [and indeed has been (Pitts 2010)] by realism about gravitational energy localization and consequently about total (gravitational + material) energy-momentum conservation. Anti-realism continues to attract philosophical adherents (Duerr 2019a, b). But because anti-realism leads to a false heuristic and realism leads to a correct heuristic in this context, there results some additional justification for taking realism about gravitational energy and conservation laws more seriously. General Relativity *loves* conservation of energy-momentum; indeed it isn’t a large exaggeration to say that General Relativity *is* energy-momentum conservation, given that the field equations and the conservation laws are logically equivalent: each entails the other (Noether 1918; Bergmann 1958; Pitts 2010, 2016a; Anderson 1967, p. 428). [This outcome charmingly satisfies Einstein’s mysterious desideratum in seeking gravitational field equations (Brading 2005; Pitts 2016b), that the gravitational field equations alone entail conservation laws, without assuming matter equations of motion.] There are doubtless other field equations than Einstein’s, such as those built with higher derivatives of the metric, with the same property but a different form of the conservation laws, so the claim that General Relativity *is* energy conservation, is something of an exaggeration. Such theories will share some important structural properties with Einstein’s theory, however. One can interpret the general relativistic objection to immaterial-to-material causation as saying that General Relativity tries hard to conserve energy and momentum (infinitely many of each, at each point in space!), much harder than other field theories do. Thus contemplation of spirit-to-matter causation sheds a bit of light on the gravitational energy localization debate in favor of realism.

Gravitational energy nonrealist Duerr has mused:

Does the use of gravitational and [gravitational wave] energy perhaps enable us to ascertain more easily GR’s empirical content? I fail to see how this could be true (Duerr 2019b).

The question of mental causation suggests, however, that it is true. Realism suggests that immaterial minds would have a harder time acting on general relativistic worlds than in non-general relativistic worlds, whereas anti-realism suggests that it would not be harder and might well be easier. What happens if a soul equipped with at most 4 scalar fields of influence $\Psi$ (and nothing else) tries to act on the physical world? The non-realist likely would think that such a soul would succeed (unless the nonrealist has done the Bianchi identity calculation), whereas the realist would wonder whether such a soul might fail. The Bianchi identities show that such a soul would indeed fail.

### Bearing on Conserved Quantity Theories of Causation

As various authors have noted, it is rather awkward or perhaps fatal for conserved quantity theories of causation that energy and momentum are no longer conserved quantities given the widespread anti-realism about conservation laws in light of General Relativity (Rueger 1998; Curiel 2000; Lam 2010; Vassallo 2020). Dowe has responded to this concern of Rueger’s as follows:

But that there are general relativistic spacetimes in which global conservation laws do not hold does not entail that global conservation laws fail in our world. Whether they do or not depends on the actual structure of spacetime, and in particular whether certain symmetries hold. As I understand it, our spacetime does exhibit the right symmetry, and that [*sic*] global conservation laws do hold in our universe as far as we know. I take it, then, that the conserved quantity theory is not refuted.

I suggested that the account holds in all physically possible worlds, that is, in all worlds which have the same laws of nature as ours. Has Rueger shown that this is not so? Not at all. To say, for example, that non-symmetric spacetimes are possible can be misleading. It means simply that it is a solution to the equations of the General Theory of Relativity. But this doesn’t mean that such a world is a physically possible world in the sense given above. If such a world violates other laws that hold in the actual world, then that world is not physically possible. This is exactly what we have in these non-symmetric spacetimes. Symmetries and conservation laws that hold in the actual world break down, so it is not a physically possible world in my sense.

Therefore we need not give up on the Conserved Quantity theory, understood as a contingent hypothesis (Dowe 2000a).

Dowe seems not to realize that maintaining such a global conservation law would be revisionary—indeed he is suggesting in effect that the usual cosmological models are not even physically possible—so the cost is greater than he envisaged. Whether a global conservation law is philosophically interesting is difficult to judge. Many philosophers are unaware that global conservation laws are a crude and archaic relative of the conservation laws that are of primary interest in physics, and hence are too easily satisfied with global conservation laws. I might also suggest that the conserved quantity theory need not be quite so contingent—it could potentially hold for all general relativistic worlds, not just those with symmetries of the geometry—if local conservation laws are employed.

If the above considerations have shown that realism about gravitational energy localization and consequently about local conservation laws in General Relativity has something going for it, then that is a boon for a conserved quantity theory of causation. If energy and momentum are no less conserved in General Relativity than in other field theories, then Rueger’s objection no longer holds just as stated. One would need to become accustomed to speaking of plural energies and momenta, however.

Does this mean that the general relativistic objection to conserved quantity theories of causation is resolved? That would be too quick.^15^ One might worry that accepting infinitely many energies (and momenta and angular momenta, for that matter) commits one to the existence of infinitely many causations, and that such a result is absurd. It seems to me, however, that either one is not committed to infinitely many causations, or that having infinitely many causations is not absurd. That is because pre-GR theories, which presumably are supposed to be unproblematic for conserved quantity theories of causation, one already had a *multiplicity* of conserved quantities, including energy (1), momentum (3), and angular momentum (3), not to mention (with different origins) charge. Did pre-general relativistic physics have at least 7 causations? If not, I do not see why infinitely many energies should imply infinitely many causations. If so, then either a multiplicity of causations is acceptable—in which case why not infinitely many if seven or eight causations are permitted—or conserved quantity theories of causation face a much broader objection than seems to have been noticed before, because one has energy-causation, x-momentum causation, z-angular-momentum causation, *etc*. already in pre-general relativistic theories On any of the three possibilities, it is not obvious that any qualitatively new problem of infinitely many causations is generated by accepting infinitely many energies in General Relativity.

There might, however, be a remnant of a problem from the original motivations for denying the reality of gravitational energy. In pre-general relativistic theories, it is difficult (except in highly symmetric situations) to transfer energy without transferring momentum, or to transfer momentum without transferring angular momentum, or the like; the various conserved quantities as indicators of causation tend to give the same result in most cases. On the other hand, it seems plausible that in General Relativity, one could find a coordinate system in which, e.g., Minkowski space-time in Cartesian coordinates up till $t=0$ is continued by Minkowski space-time in which one region has negative energy density and another region has a compensating positive energy density, still giving 0 total energy. In other worlds, it seems plausible that one can have purely coordinate-effect transfers of energy, thus giving spurious indications of causation according to a conserved quantity theory of causation.

Can a realist about gravitational energy avoid this problem? For the particular case of Minkowski space-time, arguably so: one could say that in space-times with a unique largest group of compatible symmetries [that is, such that the relevant vector fields commute, permitting the adaptation of coordinates to fit the symmetries of the space-time (Wald 1984, p. 27)], one *should* use such coordinates. Such a proposal has been made (Pitts 2010) to address Bauer’s false positive objection (Bauer 1918) that Minkowski space-time in unimodular spherical coordinates has non-zero energy density and infinite energy. Unfortunately this strategy might not work sufficiently often; already (anti)de Sitter space-times are maximally symmetric but the symmetries (Killing vectors) in many cases fail to commute, making it impossible to adapt the coordinates to as many symmetries as one might have naively hoped.

Consider the case of nearly flat static space-time with spherical symmetry—a highly symmetric situation in which nothing is happening. The admissibility of any coordinate system (a standard feature of General Relativity) and the acceptance of all the infinitely many energies (as I have proposed) suggests that one could readily find coordinates with oscillatory behavior and non-zero energy transport from one place to another, as well as coordinates that reflect the symmetry of the physical situation and no energy transport. Is there a principled way to tell a consistent and physically reasonable story, or is one stuck with intuitively spurious causation due to wavy coordinates in situations in which (by ordinary standards) nothing is happening?

Perhaps more seriously, consider a not-quite-symmetric world, such as the actual^16^ world. Due to the lack of exact symmetries, there is no coordinate that is exactly adapted to the space-time geometry. Hence it seems that nothing more than good judgment—that is, nothing principled and precise—stands in the way of admitting just any coordinates, such as the ones that might depict a significant transfer of energy from one place where basically nothing is happening to some other place. (It might be worthwhile to try to actually exhibit such coordinates; doing so in flat space-time would suffice to show that they exist more generally in weak gravitational fields.) Is there a principled way to deprecate such coordinates? One crude possibility would be to impose coordinate conditions, perhaps Fock’s harmonic coordinates (Fock 1959); but such a move seems to bludgeon the spirit of General Relativity nearly to death by giving up general covariance, rather than taking the theory as it is (as the realist aims to do). Another possibility is that extant work or further progress in making sense of approximate symmetries [on which see (Komar 1962, 1963; Matzner 1968a, b; York 1974; Taubes 1978; Zalaletdinov 1999; Dain 2004; Bona et al. 2005)] might show that in a given space-time region, some coordinates are better than others because they are closer to reflecting the space-time symmetries. Given that approximate symmetries are a mainstream problem with a strong intuitive basis, it seems fairly plausible that an adequate solution exists and might well be found.

## Conclusion

This paper has shown using coordinate-covariant calculations that General Relativity makes it harder for immaterial minds to act on the physical world, rather than being like other theories or (as one might have heard) easier than other theories. This result and other considerations might tend to show that gravitational energy is real, objectively located, and infinite in multiplicity. Such conclusions, if accepted, might be of some use in addressing a serious objection to conserved quantity theories of causation.

## Footnotes

1 Arguably General Relativity is even friendlier territory, contrary to what one hears, on which more below.

2 Strictly speaking there is still a little conservation law to violate for some models, namely, constancy of total mass-energy in asymptotically flat space-times, with a localized matter distribution (Misner et al. 1973, chs. 19, 20). Such a distribution is contrary to the usual cosmological models, though not necessarily with the facts if one is willing to suppose that the cosmos differs enough past where we can see from how it is around here (Feynman et al. 1995, p. 166; Klein 1971; Smoller and Temple 2003). Of course this conclusion might need qualification in light of the apparently nonzero value of the cosmological constant $\Lambda .$

3 It is not rare to introduce a red herring by bringing up symmetries (or the lack thereof) of the *geometry*, as though a spatio-temporally varying metric were relevant to the existence of conservation laws (Denisov and Logunov 1982; Carroll 2010; Motl 2010; Hossenfelder 2016; Physics Stack Exchange 2017; Siegel 2018; Maudlin et al. 2019). But Noether’s theorem does not know or care about geometry; it carries only about symmetries of the action (Noether 1918) or, if one permits nonvariational fields, whether the nonvariational fields have symmetries (Trautman 1966). To ask for a field with Euler–Lagrange equations to have symmetries as well is to require supererogation.

4 This point might have an analog in many people’s view of the introduction of “midichlorians” to mediate the Force in the *Star Wars* prequel (Episode I, *The Phantom Menace*): it was easy enough to play along with the Force when it was frankly magical-religious, but the introduction of an alleged scientific basis invited evaluation by different standards. There are, of course, disanalogies, such as that presumably no one believes *Star Wars* to be true.

5 Duff’s critique (Duff 1981) in terms of the failure of the expected equivalences under field redefinitions on account of mixing quantum and classical physics (each of which separately supports such equivalences) is interesting. The question of field redefinitions is considered in more detail below.

6 One might have a mental image of one vector field (locally) as a bunch of nicely layered arrows of the same length and pointing in the same direction (perhaps with components (1, 0, 0, 0)), and another vector field as bending back and forth in an irregular way with arrows of varying length (presumably with components that are complicated functions of the space-time coordinates). This distinction, however, is illusory (unless one makes a comparison to other structures): considering vectors by themselves, there is no such distinction to be made, because the two component descriptions are related by coordinate transformations (Bergmann 1958; Zajtz 1988). The difference between the uniform and non-uniform arrows *as* uniform and non-uniform (respectively), as opposed to being merely distinct vector fields, can only be maintained in relation to some other structure, perhaps the metrically flat page on which one envisages these pictures with arrows to be printed.

7 In the movie *Highlander*, the immortals (who don’t die except by violence) keep repeating, for no clearly adequate reason, that “there can be only one” as an explanation for why they are trying to kill each other.

8 Two energies are distinct because one of them is built partly out of one natural/coordinate basis $(\mathbf{\partial /\partial } x^0,$ $\mathbf{\partial /\partial } x^1$, $\mathbf{\partial /\partial } x^2,$ $\mathbf{\partial /\partial } x^3)$ related to one coordinate system, while another energy is related to a different coordinate basis $(\mathbf{\partial /\partial } x^{0^{\prime }},$ $\mathbf{\partial /\partial } x^{1^{\prime }}$, $\mathbf{\partial /\partial } x^{2^{\prime }},$ $\mathbf{\partial /\partial } x^{3^{\prime }})$ related to a different (primed) coordinate system. Thus the energies are clearly distinct, much as momentum in the *x* direction and momentum in the *y* direction are distinct in simpler theories.

9 One can perhaps find the idea in Kijowski’s work (1978) and a trajectory toward it in that of Gibbs (1997, 2010). Non-existence claims about the entire sprawling century of literature are clearly defeasible.

10 Whereas physicists happily enough utter expressions such as “conservation laws fail” or are “violated,” meaning that the continuity equation is not true, philosophers tend to find such expressions paradoxical because most think that laws must be true and violations are naughty. Thus I have attempted to avoid such expressions.

11 This collection $\Psi$ has some similarity to Lucien Hardy’s agency fields $\chi$ (Hardy 2016) in his operational approach to GR.

12 The same can be said of possible dependence of matter *u*’s transformations on the metric if *u* contains spinor fields. The nonlinear group realization formalism avoids the surplus structure of an orthonormal tetrad (10 components) and an extra local *O*(1, 3) gauge freedom (6 freedoms) to deprive the extra components of physical meaning ($16-6=10,$ the number of components of $g_{\mu \nu }$ (Seligman 1949; Ogievetsky and Polubarinov 1965b; Ogievetskiĭ and Polubarinov 1965a; Pitts 2012, 2013). The tetrad formalism is thus de-Ockhamized. A common back door from the tetrad formalism to the nonlinear realization formalism is the imposition of the symmetric tetrad gauge condition, which is very popular in studying the Einstein-Dirac system of gravity plus electrons (or the like) and in supergravity (van Nieuwenhuizen 1981; Woodard 1984; Gates et al. 1983, p. 234). One could permit the $\Psi ^A$ transformation rule to depend on matter fields *u* as well, though it is hard to see why one would.

13 One might consider the relevance of solitons (as an anonymous reader has suggested). It would seem that solitons (Manton and Sutcliffe 2004) have little relevance to this argument, because most types (except Skyrmions) either are not relevant to situations varying nontrivially in three spatial and one time dimension or involve so many fields involved that the Bianchi identities would be saturated so that there is no difficulty for the mind to act on matter. Moreover, most or all solitons are only approximately localized spatially and temporally. Merely approximate localization would imply that my soul could act on matter before Julius Caesar crossed the Rubicon (but only very weakly) and in the Andromeda Galaxy (but only very weakly), which seems absurd and which might make telekinesis and similar phenomena common.

14 One could find a fifth identity by considering electromagnetic gauge freedom and maybe a few more [perhaps irrelevant ones (Pitts 2009a; François 2019)] from the weak and strong nuclear forces. While the details might be changed, the conceptual point is not affected.

15 I thank a referee for pressing objections on this point.

16 In this section on conserved quantity theories of causation, I am not considering mental causation. Here I have in mind symmetries of the metric, not symmetries of the action as in most of the above.
